# Extreme anti-reflection enhanced magneto-optic Kerr effect microscopy

**DOI:** 10.1038/s41467-020-19724-7

**Published:** 2020-11-23

**Authors:** Dongha Kim, Young-Wan Oh, Jong Uk Kim, Soogil Lee, Arthur Baucour, Jonghwa Shin, Kab-Jin Kim, Byong-Guk Park, Min-Kyo Seo

**Affiliations:** 1 Department of Physics, KAIST, Daejeon, 34141 Republic of Korea; 2 Institute for the NanoCentury, KAIST, Daejeon, 34141 Republic of Korea; 3 Department of Materials Science and Engineering, KAIST, Daejeon, 34141 Republic of Korea

**Keywords:** Magnetic properties and materials, Imaging techniques, Magneto-optics, Nanophotonics and plasmonics, Imaging and sensing

## Abstract

Magnetic and spintronic media have offered fundamental scientific subjects and technological applications. Magneto-optic Kerr effect (MOKE) microscopy provides the most accessible platform to study the dynamics of spins, magnetic quasi-particles, and domain walls. However, in the research of nanoscale spin textures and state-of-the-art spintronic devices, optical techniques are generally restricted by the extremely weak magneto-optical activity and diffraction limit. Highly sophisticated, expensive electron microscopy and scanning probe methods thus have come to the forefront. Here, we show that extreme anti-reflection (EAR) dramatically improves the performance and functionality of MOKE microscopy. For 1-nm-thin Co film, we demonstrate a Kerr amplitude as large as 20° and magnetic domain imaging visibility of 0.47. Especially, EAR-enhanced MOKE microscopy enables real-time detection and statistical analysis of sub-wavelength magnetic domain reversals. Furthermore, we exploit enhanced magneto-optic birefringence and demonstrate analyser-free MOKE microscopy. The EAR technique is promising for optical investigations and applications of nanomagnetic systems.

## Abstract (web summary)

Magneto-optic Kerr effect microscopy is useful for dynamic magnetic studies, but is limited by the weak magneto-optical activity. Here, the authors show that extreme anti-reflection result in a Kerr amplitude as large as 20° and enables real-time detection of sub-wavelength magnetic domain reversals.

## Introduction

In fundamental studies and applications in magnetism and spintronics, it is of considerable interest to know the spatial information of magnetisation^1^. Recent discoveries of complex in-plane spin textures, such as skyrmions^2,3^, and two-dimensional ferro/anti-ferromagnetic materials^4,5^ have created new insights into and opportunities to understand strongly correlated physics and develop novel spintronic devices and applications. Scanning probe techniques, including scanning tunnelling microscopy (STM)^6^, transmission electron microscopy (TEM)^7^, magnetic force microscopy (MFM)^8^, diamond nitrogen-vacancy centre magnetometry^9^, and resonant X-ray imaging^10^, have typically been employed to investigate nano-/atomic-scale in-plane spin and magnetic textures. But, low measurement speed and limited field of view have placed many constraints on such sophisticated techniques for monitoring temporal dynamics of magnetic ordering and quasi-particles and for imaging macroscopic behaviours of magnetic domains.

**Fig. 1:**
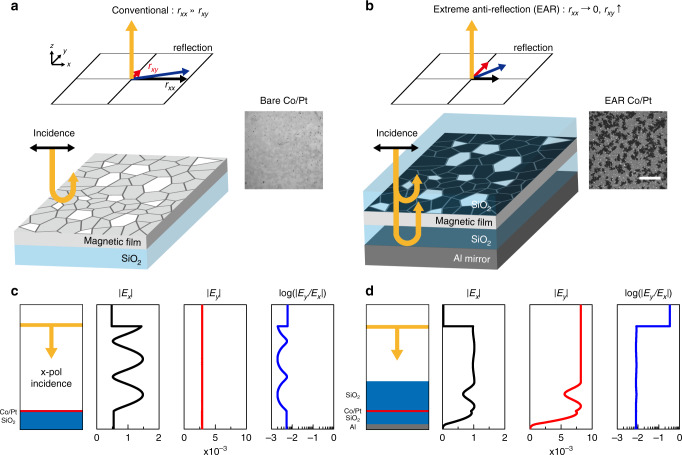
Principle of EAR-enhanced MOKE microscopy. **a**, **b** Schematic of the **a** conventional and **b** EAR-enhanced MOKE microscopy. The cooperation of the bottom Al reflector, phase-matching SiO_2_ layer, and phase-compensation SiO_2_ layer presents the magnetic material with EAR (right panel). EAR not only suppresses the non-MO reflection of the incident field (*E*_*x*_) but also enhances the generation of the MO field (*E*_*y*_). The combination of the suppressed (*r*_*xx*_) and enhanced (*r*_*xy*_) dramatically improves the performance of the MOKE microscopy. Insets, MOKE microscopy images of the bare and EAR Co/Pt films. Scale bar, 50 μm. **c**, **d** Simulated electric field amplitude profiles of the bare Co/Pt (**c**) and EAR Co/Pt (**d**) films under the normal incidence of the *x*-polarised planewave. The yellow line represents the position of the planewave source. The wavelength of light is 660 nm. The MO Kerr amplitude, the ratio between |*E*_*x*_| and |*E*_*y*_|, is plotted on the log scale.

Offering the simplest and most accessible platform for large-scale imaging, and high-speed measurement from microseconds to femtoseconds, Magneto-optical Kerr effect (MOKE) microscopy^11,12^ has exhibited irreplaceable advantages in studies of a variety of dynamic spin systems, including Barkhausen criticality, resonant magnetisation precession, ultrafast demagnetisation, and all-optical helicity-dependent switching. However, the shortcomings of MOKE microscopy are its low visibility of <10^−2^, which originates from extremely weak magneto-optical (MO) activity, and its optical diffraction limit (Fig. 1a). Engineering non-MO back-reflection, anti-reflection (AR) coatings on magnetic films have been employed to improve MOKE techniques^13–17^ and commercialise MO recording products^18^. Nonetheless, ordinary AR in the order of a few percent is still insufficient to implement high-visibility MOKE microscopy and high-precision characterisation of nanoscale magnetic domains and textures beyond the limits of conventional optics. Such advanced applications require demonstration of extreme anti-reflection (EAR) of <0.1%, which can suppress non-MO background reflection in the level of MO reflection, on the target magnetic medium. Thus, significant improvements in visibility and spatial resolution will make MOKE microscopy a pivotal and competitive technique for future research on the dynamic control and observation of novel spintronic phenomena, e.g. dynamics of nanoscale spin texture^19,20^, observation of spin accumulation^21,22^, coherent spin manipulation^23,24^, antiferro/ferrimagnetic spintronic phenomena^25–29^, and broadband spintronic terahertz emission^30,31^.

In this work, we present EAR based on an optical thin-film structure that breaks through the limits of conventional MOKE microscopy. Isolating the MO reflection from the non-MO background, EAR results in giant enhancement of the Kerr rotation, even up to 20° in a 1-nm-thin Co magnetic layer, and makes it possible to perform magnetic domain imaging with a high visibility of 0.47. Also, the enhanced magneto-optic circular birefringence allows analyser-free MOKE microscopy. Most significantly, we have demonstrated real-time measurement and statistical analysis of magnetic domain reversal beyond the optical diffraction limit and verified the power-law scalability of the Barkhausen jumps depending on domain size down to 10-nm scale.

## Results

### EAR-enhanced MOKE microscopy

At the heart of our novel high-performance MOKE microscopy is EAR, which is realised by two thin SiO_2_ spacer layers and a bottom Al mirror, as illustrated in Fig. 1b. The bottom phase-matching SiO_2_ layer enables destructive interference to suppress the non-MO reflection coefficient *r*_*xx*_. The realisation of EAR requires not only the 180° out-of-phase but also the identical amplitude of the lights reflected from the magnetic layer and the bottom Al mirror. The top SiO_2_ layer compensates for the amplitude difference between the two reflected lights and increases the completeness of EAR. In addition, the existence of the top SiO_2_ layer allows a highly confined electric field in the magnetic medium to enhance the MO reflection coefficient *r*_*xy*_. For the incident light linearly polarised (LP) along the ***x***-direction, the Kerr rotation *θ*_*K*_ and ellipticity *ε*_*K*_ are related through the Kerr amplitude |*θ*_*K*_ + *iε*_*K*_| = |tan^−1^(*r*_*xy*_*/r*_*xx*_)|. In our multilayer structure, EAR significantly enhances the Kerr amplitude, up to two orders of magnitude compared with conventional measurements. Such an extreme enhancement of the Kerr amplitude facilitates highly sensitive detection and imaging of magnetised domains (Fig. 1b). In this study, we studied the ferromagnetic/paramagnetic heterostructures (Co/Pt, Pt/Co/Pt/Ta, etc) with EAR platform, which are the most general objects in spintronics research and applications. The enhancement and isolation of the MOKE signal by EAR can also be realised, regardless of the types of magnetic media, from an atomically thin magnetic monolayer to a sub-micrometre-thick epitaxially grown magnetic film, or measurement configurations (Supplementary Note 1).

**Fig. 2:**
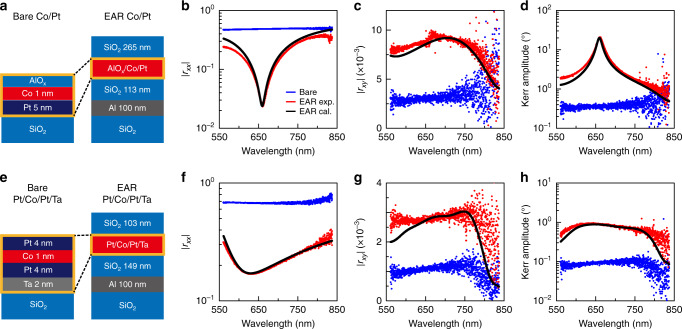
Experimental demonstration of EAR-enhanced MOKE. **a** Schematics of the bare and EAR Co/Pt layers. **b**–**d** Measured spectra of the non-MO reflection amplitude |*r*_*xx*_|, MO reflection amplitude |*r*_*xy*_|, and Kerr amplitude from the bare and EAR Co/Pt layers. **e** Schematics of the bare and EAR Pt/Co/Pt/Ta layers. **f**–**h** Measured spectra of the non-MO reflection amplitude |*r*_*xx*_|, MO reflection amplitude |*r*_*xy*_|, and Kerr amplitude from bare and EAR Pt/Co/Pt/Ta layers. We also calculated the non-MO and MO reflection amplitudes and the Kerr amplitude of the EAR Co/Pt and EAR Pt/Co/Pt/Ta films using the transfer matrix method (black solid lines).

To verify the expected advantages of EAR, we calculated the non-MO and MO reflections of a Co 1-nm/Pt 5-nm film on a SiO_2_ substrate and those for the same film embedded in an EAR multilayer (Fig. 1c, d). We assume that the magnetisation of the Co/Pt medium was saturated along the **+*z*** direction^32^ and a 660-nm-wavelength *x*-polarised planewave was normally incident from the top. The thicknesses of the top and bottom SiO_2_ layers are set to 265 nm and 113 nm, respectively, to support EAR at the target wavelength in experiments (Fig. 2). We employed the anisotropic transfer matrix method based on the Stokes vector and the Mueller matrix^33^ to calculate the birefringent magneto-optic reflection of the EAR multilayer (Supplementary Note 2). The bare Co/Pt film on the SiO_2_ substrate reflects ~23.4% of the incident light but shows a weak MO reflection of ~9.39 × 10^−4^%. On the other hand, the EAR multilayer reflects only ~5.80 × 10^−2^% of the incident light and enhances the MO reflectance to ~7.64 × 10^−3^%. As a result, the theoretical calculation predicts that the MOKE amplitude of the EAR Co/Pt film is ~20.1°, which is ~55.4 times larger than that of the bare Co/Pt film (0.363°). We note that the amplitude of non-MO electric field normalised to that of the incident wave is enhanced from 0.547 (bare Co/Pt film) to 0.920 (EAR Co/Pt film) due to the phase-compensation layer, which results in the enhancement of the MO reflectance. It is also worth noting that, in the ideal case, where the thicknesses of the top and bottom SiO_2_ layers are, respectively, 261 nm and 116 nm, the non-MO reflectance is suppressed even down to ~6.04 × 10^−6^% and a giant Kerr amplitude of ~88.4° can be realised (Supplementary Note 2).

We first experimentally demonstrate EAR-enhanced polar-MOKE for the Co/Pt film (Fig. 2a). Identical magnetic films were simultaneously deposited on a SiO_2_ substrate and a SiO_2_/Al/SiO_2_ substrate, for EAR. A 2-nm-thick AlO_*x*_ layer on the Co/Pt film was additionally deposited in situ to prevent oxidation. We achieved high-quality EAR, reaching 99.94%. Such high-quality EAR suppressed the non-MO reflection amplitude |*r*_*xx*_| down to 0.023, which was ~20 times smaller than that for the bare film (Fig. 2b). As predicted theoretically, our platform also enhanced the MO reflection amplitude |*r*_*xy*_| by 3.5 times (on average, over the examined range of wavelengths) larger than that for the bare film (Fig. 2c). In the research and applications employing the magneto-optic reflection signal itself^34,35^, the signal-to-noise ratio depends on |*r*_*xy*_|^2^ and the incident light intensity; given a noise level and incident light intensity, the signal-to-noise ratio increases ~12.25 times in intensity. The enhancement of the magneto-optic reflection originates from the highly confined electric field in the magnetic medium by the EAR structure. The combination of the enhancement of |*r*_*xy*_| and the suppression of |*r*_*xx*_| results that the Kerr amplitude in terms of tan^−1^(*r*_*xy*_*/r*_*xx*_) reached 20.1° at the EAR wavelength (660 nm), which was ~66 times larger than that of the bare Co/Pt film (0.305°) and was >10° over a wide spectral range, from 648 nm to 674 nm (Fig. 2d). We note that the Kerr amplitude determines the visibility of MOKE measurement^11^.

The enhancement and isolation of the MO signal by EAR are universal, regardless of the type of magnetic medium. We designed and fabricated an EAR multilayer structure for a Pt/Co/Pt/Ta film (Fig. 2e), which exhibits different magnetic domain reversal dynamics from the Co/Pt film. Here, although imperfect fabrication yielded relatively low-quality EAR of 97.14%, the mechanism for isolation and simultaneous enhancement of the MOKE signal was still effective; the non-MO reflectivity amplitude was suppressed by 3.98 times, while the MO reflectivity amplitude was enhanced by 2.93 times (Fig. 2f, g, respectively). Even without high-quality EAR, the Kerr amplitude reached ~0.98°, which was ~11.2 times higher than that for the bare Pt/Co/Pt/Ta film (Fig. 2h). We also experimentally confirmed the tolerance of the EAR platform to the uncertainties of layer thickness (Supplementary Note 3).

### High-visibility MOKE imaging of magnetic domains and magnetisation reversal processes

**Fig. 3:**
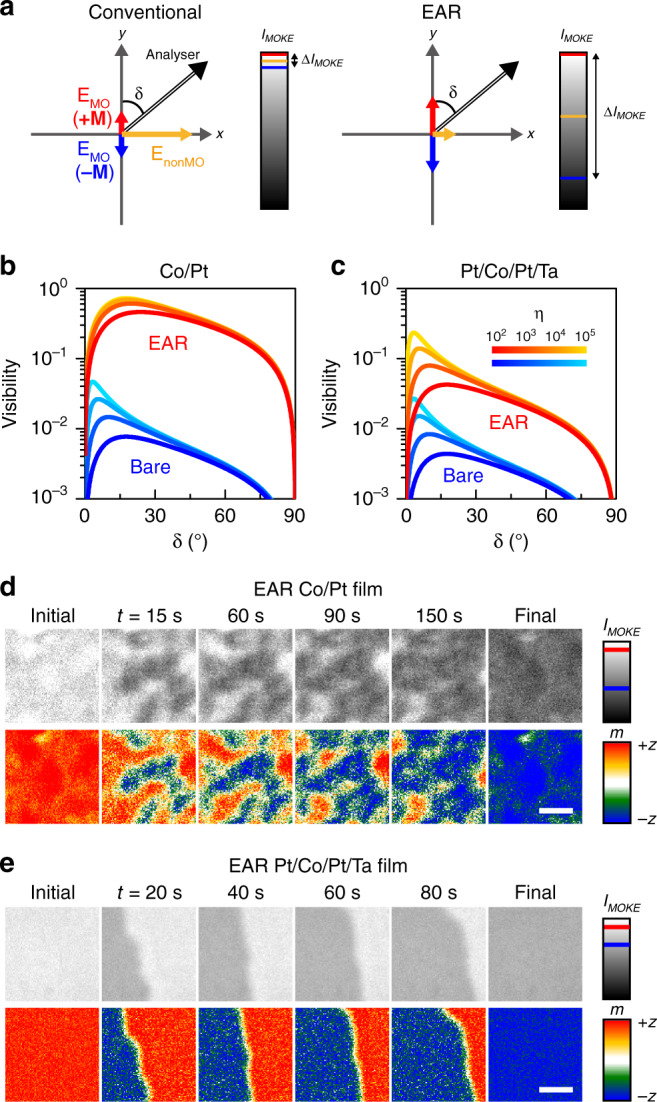
High-visibility magnetic domain imaging and high-precision magnetisation measurement. **a** Schematic of the realisation of high-visibility MOKE microscopy by EAR. The yellow arrow represents the electric field of the non-MO reflection. The red and blue arrows indicate the electric field of the MO reflection from the magnetic media of magnetisation **+*M*** and **−*M***, respectively. Here, **+*M*** (**−*M***) is the magnetisation of the fully magnetised medium in the **+*z*** (**−*z***) direction. The MOKE intensity (*I*_MOKE_) is measured using an analyser with the angle *δ* (double-lined arrow). In the colour bar, the non-MO reflection yields the reference value of *I*_MOKE_ (the yellow line) and the MO reflections of **+*M*** and **−*M*** determine the maximum and minimum of *I*_MOKE_ (the red and blue lines), respectively. Δ*I*_MOKE_, the difference between *I*_MOKE_(+***M***) and *I*_MOKE_(**−*M***), determines the available dynamic range of the employed detector and the visibility of the MOKE measurement. **b**, **c** Calculated visibility of MOKE microscopy (*V*_MOKE_) depending on the extinction ratio (*η*) and angle (*δ*) of the analyser (Supplementary Note 4). **d**, **e** Images of the measured MOKE intensity (*I*_MOKE_) and extracted magnetisation (*m*) of magnetic domain reversal, for the EAR Co/Pt (**d**) and EAR Pt/Co/Pt/Ta (**e**) films. The red and blue lines in the colour bar of *I*_MOKE_ indicate the levels of *I*_MOKE_(+***M***) and *I*_MOKE_(**−*M***), respectively. Scale bar, 3 μm.

The EAR platform significantly improves the performance of the MOKE microscopy. As illustrated in Fig. 3a, the magnetic domains with opposite vertical magnetisations (**+*M***,−***M***) exhibit different MOKE intensities (*I*_MOKE_) through an analyser of angle (*δ*). The relative phase difference between the non-MO and MO reflections can be compensated by installing a waveplate in front of the analyser^11^. The non-MO reflection determines the reference value of *I*_MOKE_ (indicated by the yellow line in colour scale of Fig. 3a), and the MO reflection increases or decreases *I*_MOKE_ (indicated by the red and blue lines in colour scale of Fig. 3a), depending on the direction of magnetisation. High-visibility MOKE measurements demand a wide intensity detection range (Δ*I*_MOKE_), ideally approaching the full dynamic range of the employed detector. Typical high-performance analysers/waveplates support high visibility for a few mrad MOKE signals, within a very narrow working range of the analyser angle. On the other hand, EAR enables high-visibility MOKE measurements over a wide range of the analyser angles, even employing a low-extinction analyser.

We derived the analytic formula for *I*_MOKE_ to estimate the performance and visibility of the EAR MOKE microscopy. We consider a vertically magnetised medium of which the magnetisation is partially saturated. Under the ***x***-polarised incidence with an electric amplitude *E*_0_, the reflected electric field is given as (*E*_*x*_, *E*_*y*_) = (*r*_*xx*_*E*_0_, *mr*_*xy*_*E*_0_). Here, *m* is the vertical magnetisation normalised to the saturation magnetisation and varies from −1 (fully magnetised to the **−*z*** direction) to +1 (fully magnetised to the **+*z*** direction). When we consider an actual analyser with a finite extinction efficiency *η*, the electric field components parallel and perpendicular to the optical axis of the analyser are given as $E_{||}\left( m \right) = r_{xx}E_0\sin \left( \delta \right) + mr_{xy}E_0\cos \left( \delta \right)$ and $E_ \bot ( m ) = \frac{1}{{\sqrt \eta }}[ {r_{xx}E_0\cos ( \delta ) -mr_{xy}E_0\sin ( \delta )} ]$, respectively. Finally, we have the MOKE intensity as follows (see more detail in Supplementary Note 4):

1 $$I_{{\mathrm{MOKE}}}\left( m \right) =	 \, | {E_{||}\left( m \right)} |^2 + \left| {E_ \bot \left( m \right)} \right|^2 \\ =	 \, \left| {E_0} \right|^2\left[ {| {r_{xx}\sin \left( \delta \right) + mr_{xy}\cos \left( \delta \right)} |^2 + \eta ^{ - 1}| {r_{xx}\cos \left( \delta \right) - mr_{xy}\sin \left( \delta \right)} |^2} \right]$$

Figure 3b, c shows the calculated visibilities of MOKE measurements for the bare and EAR films, respectively, depending on the extinction efficiency and the analyser angle (Supplementary Note 5). For the bare Co/Pt film, although a high-extinction analyser (*η* = 10^5^) was used, the visibility was only ~0.047. On the other hand, the EAR Co/Pt film, with the high-extinction analyser, showed a high MOKE visibility of 0.72 at the peak and values >0.10, over a wide range of the analyser angle, from 1.2° to 79°. Besides, the use of a low-extinction analyser (*η* = 10^2^) still supports the high visibility of 0.46 at the peak and values >0.10, for the analyser angle in the 2.5°–78° range, which still exceeds the values obtained using the high-extinction analyser for the bare Co/Pt film. Despite its imperfect performance, the EAR Pt/Co/Pt/Ta film’s visibility reaches 0.23 and 0.042, for the high- and low-extinction analysers, respectively.

We demonstrated high-visibility MOKE imaging of two representative magnetisation reversal processes in ferromagnetic media: domain nucleation (annealed Co/Pt film, Fig. 3d) and domain wall propagation (non-annealed Pt/Co/Pt/Ta film^36,37^, Fig. 3e). In the experiment, all of the magnetic domains of the 1-nm-thin Co medium were magnetised initially in the **+*z*** direction, and then an external magnetic field (320 G for Co/Pt, 100 G for Pt/Co/Pt/Ta) in the −***z*** direction was applied to trigger magnetisation reversal. For the Co/Pt film, EAR MOKE imaging under a 660 ± 5 nm light-emitting diode illumination resolves the magnetic domains with opposite vertical magnetisations, with high visibility of 0.47 on average over the field of view. The angle and extinction ratio of the employed analyser were 10° and ~10^3^, respectively. The combination of the high visibility and the large analyser angle increases the accuracy of solving the quadratic equation for extracting vertical magnetisation from the MOKE intensity data (Supplementary Note 4). We were able to determine not only the direction but also the magnitude of the net vertical magnetisation of the magnetic domains within the resolution area of the objective. In the EAR Pt/Co/Pt/Ta film, the domain wall propagation, showing irregular development owing to the Barkhausen jumps, was clearly measurable, with high visibility of ~0.14. Note that, under the same measurement conditions, the visibility of the bare Co/Pt (Pt/Co/Pt/Ta) film was only ~0.025 (~4.6 × 10^−4^) (Supplementary Note 6).

### Real-time measurement and statistical analysis of magnetic domains beyond the diffraction limit

**Fig. 4:**
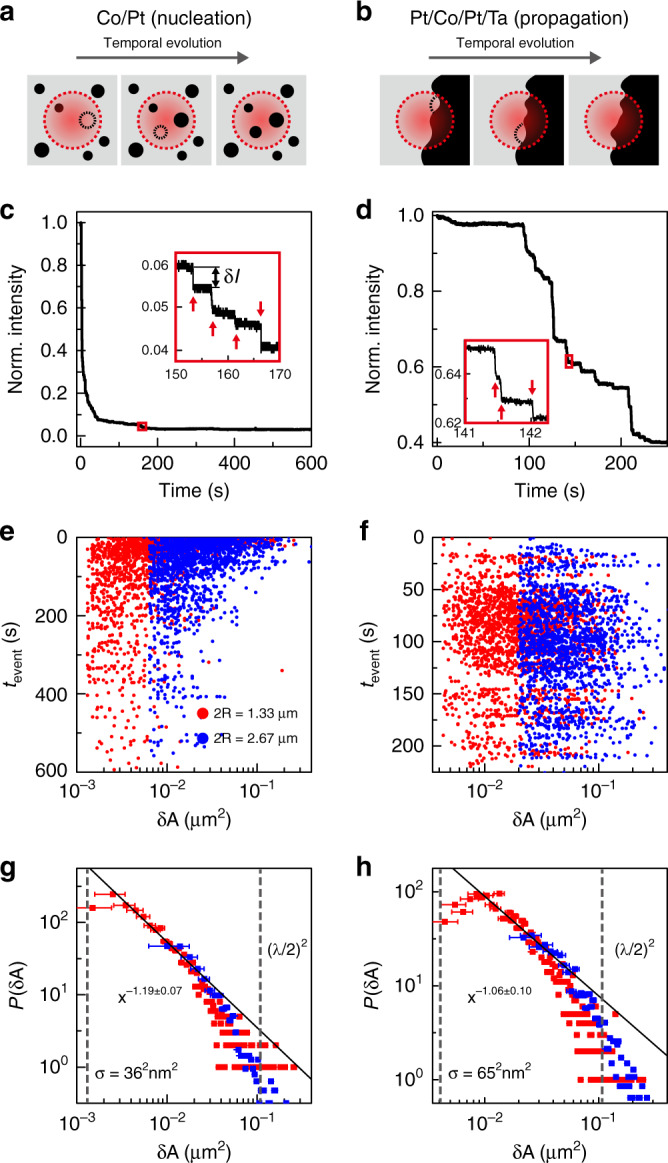
Barkhausen jumps beyond the diffraction limit. **a**, **b** Barkhausen jumps, the magnetisation reversals of individual coherent magnetic domains, cause abrupt changes of the MOKE intensity. The dotted red circle indicates the area of detection. The **a** Co/Pt and **b** Pt/Co/Pt/Ta films exhibit reverse domain nucleation and domain wall propagation, respectively. **c**, **d** Real-time measurements of magnetisation reversals of the **c** EAR Co/Pt and **d** EAR Pt/Co/Pt/Ta films. As shown in the magnified plots (insets), the Barkhausen jumps far below the wavelength scale (red arrows) are successfully resolved in terms of the MOKE intensity change (*δI*). **e**, **f** Temporal statistics of the Barkhausen jumps depending on the size (*δA*) in the EAR Co/Pt (**e**) and EAR Pt/Co/Pt/Ta (**f**) films, respectively. The measurements with different diameters of the region of interest (*2R*) of 1.33 μm and 2.67 μm are plotted by the red and blue dots, respectively. **g**, **h** Distribution of the Barkhausen size (*δA*) in EAR Co/Pt (**g**) and EAR Pt/Co/Pt/Ta (**h**), respectively. The scale-invariant Barkhausen jump events are fitted by a power law for the domain size (solid line). The vertical dashed lines indicate the minimal size of measurable domains (*σ*) and the optical diffraction limit ((*λ*/2)^2^). The error bars represent the signal detection resolution of the measurement setup (Methods and Supplementary Note 8).

The most innovative result of this study is the optical measurement of the statistics of the Barkhausen jumps^38,39^ in the sub-wavelength regime. As illustrated in Fig. 4a, b, the intensity of the reflected MOKE signal changes depending on the area ratio of the opposite magnetisation domains inside the detection area (indicated by the red dotted circle). In real-time measurements, the Barkhausen jumps are observed in the form of abrupt stepwise changes of the MOKE intensity (Fig. 4c, d). We converted the stepwise intensity change (*δI*) to the area of the reversed magnetic domain (*δA*) based on the quadratic equation. The employed analyser angles were 20° and 10° for the EAR Co/Pt and Pt/Co/Pt/Ta films, respectively. It is however notable that, when employing a large analyser angle, the MOKE intensity changes almost linearly with the reversed domain area, as *δI*/Δ*I*_MOKE_ ≅ *δA*/*πR*^2^. Here, Δ*I*_MOKE_ is the MOKE intensity change when all magnetic domains in the detection area of *πR*^2^ are reversed completely. In experiments, a 660-nm-wavelength laser illuminated the samples, and a pinhole cropped the signal from an area with the diameter (2*R*) of 1.33 μm and 2.67 μm. The incident laser power was ~110 μW, sufficiently weak to avoid thermal effects but sufficiently strong to use almost the full dynamic range of the employed detector (Supplementary Note 7). The detection noise fluctuation, which is the resolution limit of the MOKE intensity change measurement, determines the minimal size of measurable domains (*σ*). In our experimental setup, the output power fluctuation of the employed laser diode dominates in the signal fluctuation (Supplementary Note 8). Owing to its high visibility, the EAR MOKE measurement produced a significantly large difference of the electric signal from the detector between the positive (**+*z***) and negative (**−*z***) saturation (Supplementary Note 9) and enabled to resolve the magnetisation reversal of domains with areas as small as ~1.28 × 10^−3^ μm^2^ (~4.27 × 10^−3^ μm^2^) in the Co/Pt (Pt/Co/Pt/Ta) film, beyond the optical diffraction limit.

We temporally recorded the Barkhausen jump events depending on the area of the reversed magnetic domain (Fig. 4e, f). We plotted 3549 (3705) events for the EAR Co/Pt (Pt/Co/Pt/Ta) film obtained from 40 real-time measurements. The measurement of the size-dependent dynamics of magnetisation reversal reveals the distinction between the domain nucleation and domain wall propagation processes. As shown in Fig. 4e, the dynamics of domain nucleation exhibits temporal exponential decay, with the reaction rate *k*. The Arrhenius equation gives $k \propto {\mathrm{exp}}( - E_a/k_{\mathrm{B}}T) = \exp \left( {\left( {K_{\mathrm{u}} - {\boldsymbol{M}} \cdot {\boldsymbol{B}}} \right)V/k_{\mathrm{B}}T} \right)$, where *K*_u_, ***M · B***, and *V* are the perpendicular magnetic anisotropy constant, the Zeeman energy density, and the volume of the magnetic domain, respectively^36^. On the other hand, in the domain wall propagation, interactions between nearest-neighbour domains dominate; thus, the magnetisation reversal is independent on the domain size (Fig. 4f). It is well known that the Barkhausen jumps satisfy power-law scaling of domain sizes^40^. In this work, we for the first time verify the power-law scalability down to the 10 nm scale, which has not been demonstrated yet using conventional optical techniques (Fig. 4g, h). The critical exponents for the examined Co/Pt and Pt/Co/Pt/Ta films were 1.19 ± 0.07 and 1.06 ± 0.10, respectively. These values are consistent with those of similar Co-based systems studied by X-ray microscopy^41^. The observed critical exponents reflect that the magnetic films employed in this work are in a universality class where short-range domain wall surface tension is dominant^39^. Although it is an indirect measurement method, the EAR platform enabled us to quantify the movement of magnetic domain walls as small as tens of nanometres and analyse Barkhausen jumps beyond the optical diffraction limit. Fast and highly accessible optical methods can be a good alternative, when the methods for direct spatial observation of individual magnetic domains or domain wall segments, such as transmission electron microscopy^7^, scanning probe microscopy^9^, and X-ray imaging^36^, are not available. We expect that using high-performance photodetectors with a large dynamic range and GHz-level bandwidth will allow further investigations into the size-dependent dynamics of magnetic domain reversal and domain-wall creep motion^42,43^ on the atomic scale and with the nanosecond-scale speed.

### Enhanced MO birefringence and analyser-free MOKE microscopy

**Fig. 5:**
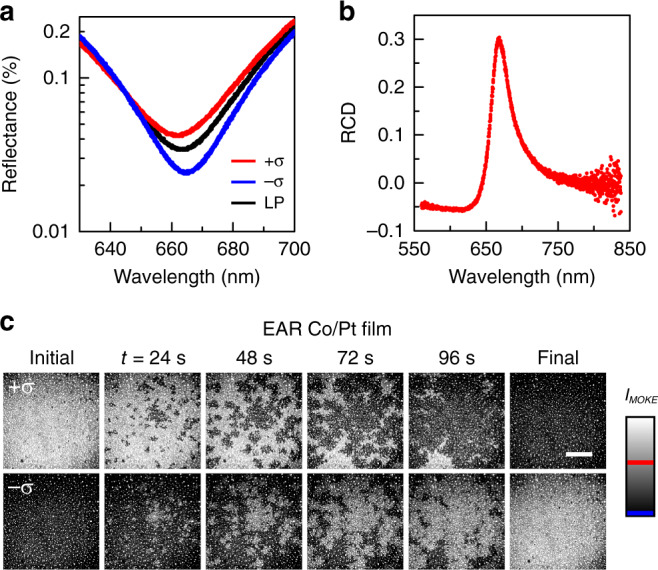
Analyser-free MOKE microscopy. **a** Measured reflectance spectra of the EAR Co/Pt film under the right- (−*σ*) and left-handed (+*σ*) circularly polarised incidence and the LP incidence. **b** Spectrum of the RCD. **c** Measured analyser-free MOKE microscope images of magnetisation reversals of the EAR Co/Pt film under the left- and right-handed circularly polarised illuminations. The red and blue lines in the colour bar indicate the maximal and minimal values of the measured MOKE intensity, respectively. Scale bar, 50 μm.

The isolation of MO reflection enables analyser-free MOKE microscopy. The MO birefringence causes the differential reflectance of left- and right-hand circularly polarised light. In the Co medium in this study, the domain magnetised in the **+*z*** direction reflected left-hand circularly polarised light more than right-hand circularly polarised light, and vice versa. As shown in Fig. 5a, the MO birefringent reflection emerges from the non-MO reflection near the EAR condition. The ratio of the reflectance of the right- and left-handed circularly polarised light was up to ~1.9, corresponding to the reflection circular dichroism (RCD) of ~0.30 (Fig. 5b), which is defined as RCD = (*R*_LCP_ − *R*_RCP_)/(*R*_LCP_ + *R*_RCP_). Such a high circular birefringence enables straightforward but powerful MOKE microscopy without any analyser or waveplate. The bright-field microscopy, using only circularly polarised illumination, excellently resolved the regions magnetised in the **+*z*** and **−*z*** directions (Fig. 5c). Analyser-free MOKE detection can also facilitate the accessibility of MO devices.

## Discussion

In summary, EAR provides a simple, promising way for significantly expanding the conventional limits of MOKE microscopy. High-visibility MOKE microscopy based on the remarkably enhanced Kerr amplitude enables real-time measurement and statistical analysis of nanoscale magnetic domain reversal beyond the optical diffraction limit. In the future, we expect to use the EAR MOKE microscopy approach for investigating state-of-the-art topics in the field of magnetism and spintronics, such as skyrmions^19,44^, magnons^45^, and magnetic solitons^46,47^. We also believe that the EAR technique will significantly facilitate utilisation of optically functional nanoscale-thin films, from semiconductor quantum wells^48^ to two-dimensional materials (CrI_3_, CrBr_3_, and FePS_3_)^49–51^, for novel photonic and electro-/magneto-optic devices and systems^52–54^.

## Methods

### Fabrication

The 100-nm-thick Al mirror layer for EAR was deposited by the conventional electron beam evaporation. The phase-matching and phase-compensation SiO_2_ layers were deposited by the radio-frequency sputtering process. We employed physical vapour deposition by sputtering to form the Co/Pt and Pt/Co/Pt/Ta layers. The magnetic layers of the bare and EAR samples were grown at the same time. To support the reverse domain nucleation behaviour, we performed post-annealing treatment on the Co/Pt layer at 350 °C.

### Measurement

The fabricated samples were mounted on a precise, non-magnetic XYZ translation stage at room temperature. A permanent neodymium magnet with a magnetic field strength of ~5000 G at the surface was employed to apply an external magnetic field along the direction normal to the magnetic layer. A motorised translation stage moved the magnet and controlled the strength of the magnetic field applied to the target sample, up to 5000 G. Employing a Hall effect sensor (Hirst Magnetic Instruments GM08), we mapped the direction and strength of the magnetic field depending on the distance relative to the magnet. In spectral measurements, a broadband light-emitting diode (*λ* = 640 nm, Δ*λ* = ±50 nm) illuminated the sample, and a spectrometer (Princeton Instruments Acton SP2300) and high-performance charge-coupled device (Princeton Instrument PIXIS-100BR) were used. Here, to obtain a planewave-like illumination, we used a × 5 objective lens with a low numerical aperture (NA) of 0.1. In MOKE imaging, a narrowband light-emitting diode (LED) (*λ* = 660 nm, Δ*λ* = ±5 nm) illuminated the target sample, and a high bit-depth complementary metal-oxide-semiconductor (CMOS) camera (Sony IMX249LLJ-C) was used. For the Barkhausen jump measurements, a 660-nm-wavelength laser diode, femtowatt photoreceiver (Newport 2151), and analog-to-digital converter (National Instruments PCI-6111) were used. We used a × 50 long-working-distance objective lens (Mitutoyo, NA 0.42) for MOKE imaging and for the Barkhausen jump measurements. The dynamic range and linear responsivity of the photodetector were ~4.65 × 10^3^ and ~1.06 × 10^6^ V W^−1^, respectively (Supplementary Note 10).

### Theoretical calculations

The permittivity of the MO medium for theoretical calculations was modelled by a Hermitian tensor, consisting of the diagonal non-magnetic permittivity and off-diagonal magnetic components^55^. The non-magnetic permittivity of the Co/Pt and Pt/Co/Pt/Ta media was obtained from ellipsometry measurements of the bare films (*ε*_Co/Pt_ = −11.9 + 18.1i, *ε*_Pt/Co/Pt_ = −13.6 + 24.6i at *λ* = 660 nm). The off-diagonal magnetic components were extracted from the MOKE measurements of the bare films (*Q*_Co/Pt_ = 3.83 × 10^−3^ + 1.48 × 10^−2^i, *Q*_Pt/Co/Pt_ = 9.22 × 10^−5^ + 5.23 × 10^−3^i at *λ* = 660 nm). The permittivity of the bare films was then used to calculate the reflection amplitude (*r*_*xx*_, *r*_*xy*_) spectra and MOKE spectra, using the anisotropic transfer matrix method^33^ (Fig. 2). The permittivity of the deposited SiO_2_ layer was also obtained from the ellipsometry measurements (*ε*_SiO2_ = 2.16 at *λ* = 660 nm). We used the permittivity of Al from experimental measurements^56^ (*ε*_Al_ = −60.8 + 25.7i at *λ* = 660 nm). The electric field profiles (Fig. 1c, d) were calculated using the finite-difference time-domain (FDTD) simulation (Lumerical Solutions, Inc). We used the grid attribute technique of the employed FDTD calculator to deal with the Hermitian permittivity tensor of the magnetic medium.

## Supplementary information

Supplementary Information

## Data Availability

The data that support the findings of this study are available from the corresponding author upon reasonable request.
