# Supplementary material for: Extreme anti-reflection enhanced magneto-optic Kerr effect microscopy
Source: Nat Commun. 2020 Nov 23;11:5937. doi: 10.1038/s41467-020-19724-7 (PMC7684307; doi:10.1038/s41467-020-19724-7)
Supplement: Supplementary file 1 — Supplementary Information [file 41467_2020_19724_MOESM1_ESM.pdf]

Supplementary Information

Extreme anti-reflection enhanced magneto-optic Kerr effect microscopy

Dongha Kim[1,3], Young-Wan Oh[2,3], Jong-Uk Kim[2,3], Soogil Lee[2,3], Arthur Baucour[2,3], Jonghwa Shin[2,3], Kab-Jin Kim[1], Byong-Guk Park[2,3], and Min-Kyo Seo[1,3*]

[1]Department of Physics, KAIST, Daejeon 34141, Republic of Korea,

[2]Department of Materials Science and Engineering, KAIST, Daejeon 34141, Republic of Korea,

[3]Institute for the NanoCentury, KAIST, Daejeon 34141, Republic of Korea,

*Corresponding author: minkyo_seo@kaist.ac.kr

Supplementary Note 1. Universality of EAR-assisted MOKE enhancement

Extreme anti-reflection (EAR) employment in magnetic material is universal. It can be achieved regardless of the wavelength of light, the complex refractive index and thickness of the magnetic layer. We first examine the wavelength of EAR for the AlO_x 2 nm / Co 1 nm / Pt 5 nm film depending on the thicknesses of the phase-matching (bottom) and phase-compensation (top) SiO_2 layers. The incident light is polarised along the x-axis. As shown in Supplementary Figure 1, EAR can be achieved at different target wavelengths over a wide spectral range from the visible to near-infrared region. Suppressing the non-magneto-optic (non-MO) reflection amplitude r_{xx} and enhancing the MO reflection amplitude r_{xy} are successfully achieved for every target wavelengths. In addition, the Kerr amplitude $\left|\tan^{-1}(r_{xy}/r_{xx})\right|$ is close to the theoretical limit of 90°. Detailed information on the thicknesses of the phase-matching and phase-compensation layers for the given target wavelengths is shown in Supplementary Table 1.

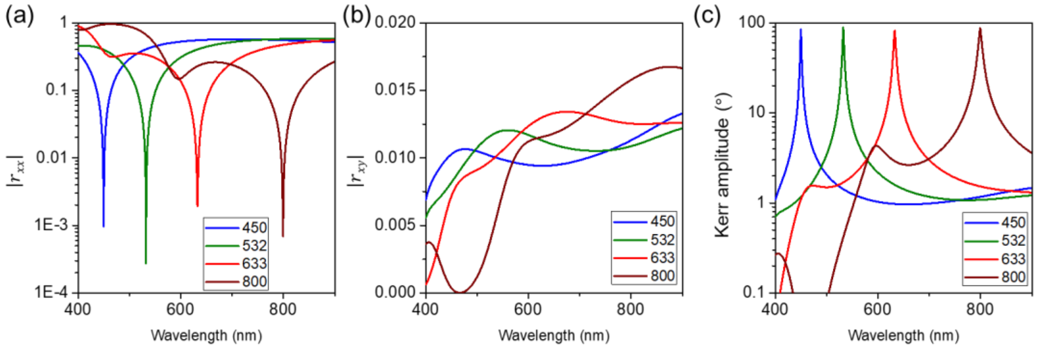

Supplementary Figure 1 EAR-assisted MOKE enhancement in the Co/Pt film for different target wavelengths of 450, 532, 633, and 800 nm. Calculated spectra of (a) non-MO reflection amplitude $|r_{xx}|$, (b) MO reflection amplitude $|r_{xy}|$, and (c) Kerr amplitude ($\left|\tan^{-1}(r_{xy}/r_{xx})\right|$).

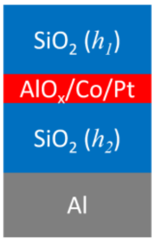

Target wavelength (nm)	h_1 (nm)	h_2 (nm)
450	158	76
532	201	90
633	252	108
800	337	139

Supplementary Table 1 Thickness information of EAR Co/Pt for different target wavelength of 450, 532, 633, 800-nm.

Second, we present that the EAR technique can work universally on a variety of magnetic media. As shown in Supplementary Figure 2, we were able to demonstrate EAR and Kerr amplitude enhancement for 10-nm-thick Ni, Co, and Fe films. Here, the target wavelength is 660 nm. The spectral behaviours of EAR and Kerr amplitude are almost the same regardless of the type of magnetic medium. Detailed information on the thicknesses of the phase-matching and phase-compensation layers for the examined magnetic media is shown in Supplementary Table 2.

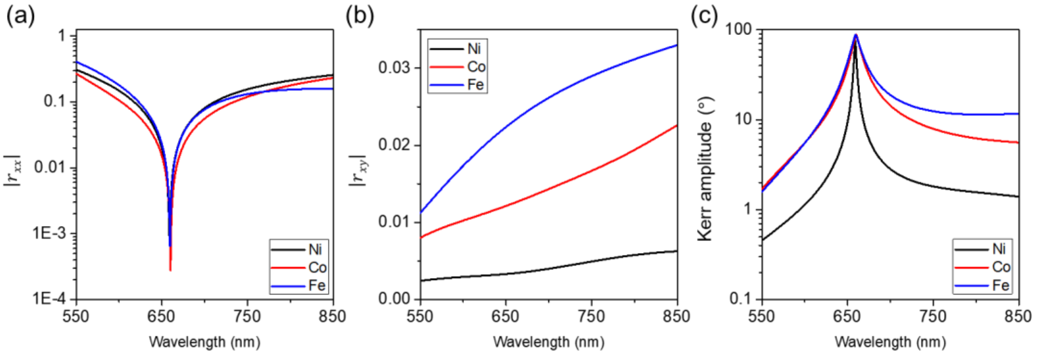

Supplementary Figure 2 EAR-assisted MOKE enhancement in 10-nm-thick Ni, Co, and Fe films. Calculated spectra of (a) non-MO reflection amplitude $|r_{xx}|$, (b) MO reflection amplitude $|r_{xy}|$, and (c) Kerr amplitude ($|\tan^{-1}(r_{xy}/r_{xx})|$).

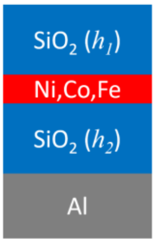

Target material	h_1 (nm)	h_2 (nm)
Ni	68	125
Co	88	138
Fe	138	131

Supplementary Table 2 Thickness information of EAR Co/Pt for different target materials of Ni, Co, Fe.

Third, we examined the utilization of the extreme anti-reflection (EAR) platform for the following cases: the atomically thin Co film with a thickness of 0.3 nm and the bulk Co substrate (see Supplementary Figure 3 below). We employed a SiO2/Pt/SiO2 multilayer to support EAR for both cases, as shown in Supplementary Figs. 3(a) and 3(e). First, the atomically thin Co monolayer film placed on top of the EAR multilayer exhibits an extremely enhanced Kerr amplitude up to ~80 degrees at the target wavelength (660 nm), as shown in Supplementary Fig. 3(d). We note that such a configuration, in which the atomically thin magnetic layer is placed on the top of the EAR multilayer, is highly promising for utilizing the compelling magnetic two-dimensional (2D) materials, including CrI_3, $CrBr_3$, and $FePS_3$ [1-3]. Second, the bulk Co substrate beneath the EAR multilayer can also have an enhanced Kerr amplitude up to ~62 degrees at the target wavelength (Supplementary Fig. 3(h)). The SiO_2/Pt/SiO_2 multilayer provides not only the 180-degree out-of-phase but also the identical amplitude required for realising EAR.

Fourth, we examined the applications of the extreme anti-reflection (EAR) platform to the magnetic film with a small Voigt constant (see Supplementary Fig. 3(i)-3(k)). First, we suppose a 10-nm-thick Co layer with a smaller Voigt constant with a factor of ξ, which varies from 10^0 to 10^{-2}, than the natural Co layer, keeping its non-magneto-optic permittivity. The weakly-magnetised Co film is coupled with two thin SiO_2 spacer layers and a bottom Al mirror, as similar to the cases of the Co/Pt and Pt/Co/Pt/Ta media in the main manuscript. At the target wavelength (660 nm), the magnetic film with a Voigt constant smaller than 10 times (100 times) than the natural Co layer exhibits a Kerr amplitude enhanced up to as large as 79.1 (27.4) degrees, as shown in Supplementary Fig. 3(k). On the other hand, as shown in Fig. S3(j), the bare magnetic layer, of which the Voigt constant is 10 times (100 times) smaller than the natural Co layer, gives the Kerr amplitude of only about 4.54×10^{-2} degrees (4.55×10^{-3} degrees), which the MOKE microscope with a common configuration hardly resolves.

Fifth, we examined a 300-nm-thick YIG film on the GGG substrate, as shown in Supplementary Figs. 3(l)-(o). The refractive index of the YIG film and GGG substrate is 2.391 and 1.961 at the wavelength of 660 nm, respectively. Supplementary Figs. 3(l)-(o) clearly indicates that the EAR-enhanced MOKE technique is applicable even in thick ferromagnetic layers having the specific substrate. As the sub-micrometre-thick YIG films are widely used in spintronic research, in particular for magnonic device applications [4], this result demonstrates that our technique can be applied in various research areas with less restriction. We clarify that the main target of our study is to present an advanced optical platform for investigating heterostructure films of magnetic transition metals (e.g., Co, Fe, and Ni) and paramagnetic metals (e.g., Pt, Pd, Ir, and Ta) deposited by sputtering process. This is because those heterostructures are the core magnetic systems not only in academic researches like spin orbit torque studies [5] but also in industrial applications such as magnetic random-access memory (MRAM) [6]. It is known that the sputtered magnetic films are rather compatible with arbitrary substrates. For epitaxial magnetic films, layer transfer and wafer bonding techniques can bring a chance to employ extreme anti-reflection. For example, the transfer of an epitaxial yttrium iron garnet (YIG) layer without notable quality degradation from the gadolinium gallium garnet (GGG) substrate to the Si substrate has been demonstrated [7,8]. If a specific bottom substrate for epitaxial growth can never be removed, a similar approach to the simulations in Supplementary Figs. 3(e) and 3(l) can be employed.

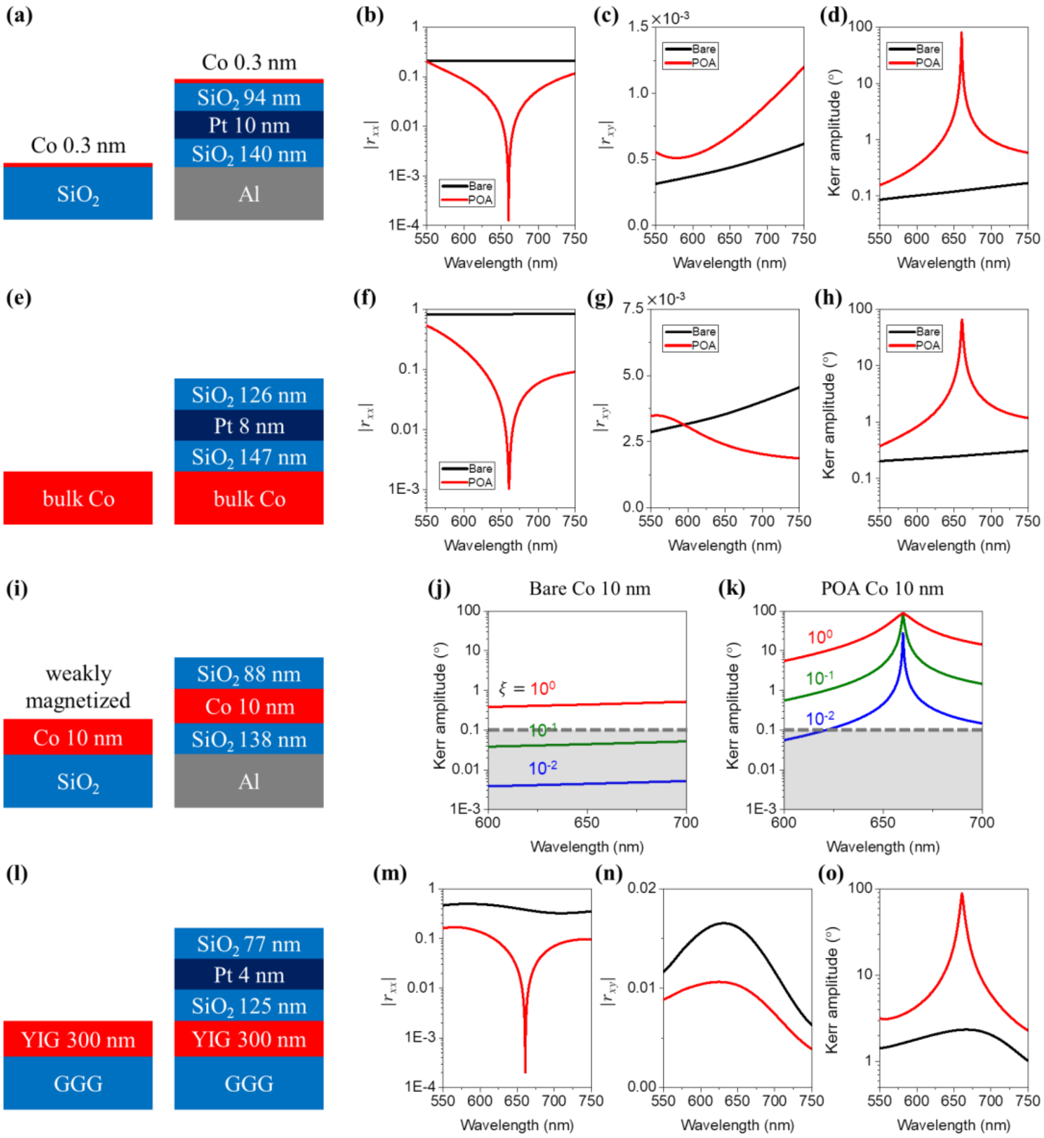

Supplementary Figure 3 (a) Schematics of the bare and EAR Co (0.3 nm) layers. (b-d) Calculated spectra of non-MO reflection amplitude $|r_{xx}|$, MO reflection amplitude $|r_{xy}|$, and Kerr amplitude $\tan^{-1}(|r_{xy}/r_{xx}|$. (e) Schematics of the bare and EAR bulk Co substrates. (f-h) Calculated spectra of non-MO reflection amplitude, MO reflection amplitude, and Kerr amplitude. (i) Schematics of the bare and EAR Co (10 nm) layers. The Co layer is supposed to be weakly magnetised with a smaller Voigt constant with a factor of ξ than the natural Co layer. Calculated Kerr amplitude spectra of the bare Co (j) and EAR Co (k) films depending on the magnetisation ratio. (l) Schematics of the bare and EAR YIG 300 nm on GGG substrate. (m-o) Calculated spectra of non-MO reflection amplitude, MO reflection amplitude, and Kerr amplitude.

Finally, we considered to apply the EAR platform to the media with in-plane magnetisation in terms of the longitudinal-MOKE (l-MOKE) and transverse-MOKE (t-MOKE) measurements (Supplementary Figure 4). Here, we examined a 10-nm-thick magnetic layer of which the optical permittivity is identical to the natural Co layer. l-MOKE and t-MOKE, in general, have amplitudes one order smaller than p-MOKE and require oblique incidence of light. We optimised the EAR multilayers for the incidence with an angle of 60 degrees. For l-MOKE, the EAR Co multilayer suppresses the non-MO reflection amplitude down to 4.76×10^{-4} and thus exhibits an extremely enhanced Kerr amplitude up to ~44.5 degrees at the target wavelength (660 nm), as shown in Supplementary Figs. 4(b)-(d). For t-MOKE, the reflectance modulation depending on the direction of the magnetisation is typically measured. As shown in Supplementary Figs. 4(f)-(h), the EAR multilayer increases the reflectance modulation depth up to ~24.7%, which is ~42.0 times larger than that of the bare layer (0.588%). The reflectance modulation depth is defined as the difference of the reflectance between the magnetic layers magnetised in the **+x** and **–x** directions normalised to the reflectance of the non-magnetised film.

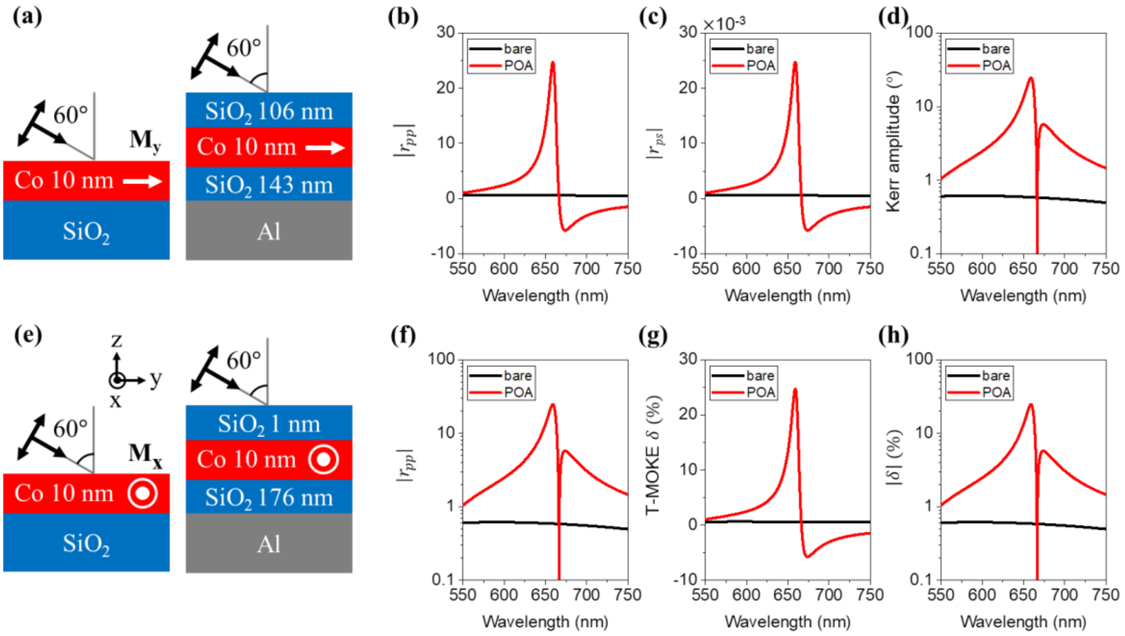

Supplementary Figure 4 (a) Schematic of the application of EAR to l-MOKE measurement. (b-d) Calculated spectra of the non-MO reflection amplitude, MO reflection amplitude, and l-MOKE amplitude from the bare and EAR Co layers. (e) Schematic of the application of EAR to t-MOKE measurement. (f-h) Calculated spectra of the non-MO reflection amplitude, t-MOKE reflectance modulation ratio $|\delta|$, and its amplitude from the bare and EAR Co layers.

Supplementary Note 2. Optimization of the phase-matching and phase-compensation layers

We employed the anisotropic transfer matrix method based on the Stokes vector and the Mueller matrix [9] to calculate the birefringent magneto-optic reflection of the EAR multilayer. The 4×4 transmission matrix (M-matrix) contains all possible light reflection and transmission routes inside the multilayer structure. By decomposing the M-matrix into four 2×2 submatrices (*G*, *H*, *I*, and *J*), we can calculate the Fresnel reflection coefficients (r_{pp}, r_{ps}, r_{sp}, and r_{ss}) as follows:

$$M = \begin{pmatrix} G & H \\ I & J \end{pmatrix}, \qquad \begin{pmatrix} r_{ss} & r_{sp} \\ r_{ps} & r_{pp} \end{pmatrix} = IG^{-1}$$

The M-matrix is given by the serial multiplication of the boundary matrix (A_j) and the propagation matrix (D_j) of the *j*th layer. Employing A_j and D_j of the six optical layers, (1) Air superstrate, (2) SiO_2, (3) AlO_x/Co/Pt or AlO_x/Pt/Co/Pt/Ta, (4) SiO_2, and (5) Al, we can represent the M-matrix as

$$M = A_1^{-1}(A_2D_2A_2^{-1})(A_3D_3A_3^{-1})(A_4D_4A_4^{-1})A_5$$

When the *yz*-plane is the incident plane, the boundary matrix A_j is given as

$$A_j \equiv \begin{pmatrix} 1 & 0 & 1 & 0 \\ \frac{iQ_j\alpha_{yj}^2}{2}\left(m_y\frac{1+\alpha_{zj}^2}{\alpha_{yj}\alpha_{zj}} - m_z\right) & \alpha_{zj} & -\frac{iQ_j\alpha_{yj}^2}{2}\left(m_y\frac{1+\alpha_{zj}}{\alpha_{yj}\alpha_{zj}} + m_z\right) & -\alpha_{zj} \\ -\frac{in_jQ}{2}\left(m_y\alpha_{yj} + m_z\alpha_{zj}\right) & -n_j & -\frac{in_jQ_j}{2}\left(m_y\alpha_{yj} - m_z\alpha_{zj}\right) & -n_j \\ n_j\alpha_{zj} & -\frac{in_jQ_j}{2}\left(m_y\frac{\alpha_{yj}}{\alpha_{zj}} - m_z\right) & -n_j\alpha_{zj} & \frac{in_jQ_j}{2}\left(m_y\frac{\alpha_{yj}}{\alpha_{zj}} + m_z\right) \end{pmatrix}$$

Here, Q_j and n_j are the Voight parameter and the refractive index of the *j*th layer, respectively, and $\vec{m} = (m_x, m_y, m_z)$ is the normalized magnetisation. $\alpha_{yj} = \sin\theta_j$ and $\alpha_{zj} = \cos\theta_j$, where θ_j is the complex refractive angle with respect to the **z**-axis in the *j*th layer determined by Snell's law. The propagation matrix D_j is given as

$$D_j \equiv \begin{pmatrix} U\cos\delta^i & U\sin\delta^i & 0 & 0 \\ -U\sin\delta^i & U\cos\delta^i & 0 & 0 \\ 0 & 0 & U^{-1}\cos\delta^r & U^{-1}\sin\delta^r \\ 0 & 0 & -U^{-1}\sin\delta^r & U^{-1}\cos\delta^r \end{pmatrix}$$

where $U = \exp\left(-i\frac{2\pi}{\lambda}n_j\alpha_{zj}d_j\right)$, $\delta^i = -\frac{\pi n_jQd_jg^i}{\lambda\alpha_{zj}}$, $\delta^r = -\frac{\pi n_jQd_jg^r}{\lambda\alpha_{zj}}$, $g^i = m_z\alpha_{zj} + m_y\alpha_{yj}$, and $g^r = m_z\alpha_{zj} - m_y\alpha_{yj}$. For the normal incidence ($\theta_j = 0$), the boundary and propagation matrices are simplified as

$$A_j = \begin{pmatrix} 1 & 0 & 1 & 0 \\ 0 & 1 & 0 & -1 \\ -\frac{in_jQ}{2}m_z & -n_j & \frac{in_jQ_j}{2}m_z & -n_j \\ n_j & \frac{in_jQ_j}{2}m_z & -n_j & \frac{in_jQ_j}{2}m_z \end{pmatrix}$$

$$\text{and } D_j = \begin{pmatrix} U\cos\delta^i & U\sin\delta^i & 0 & 0 \\ -U\sin\delta^i & U\cos\delta^i & 0 & 0 \\ 0 & 0 & U^{-1}\cos\delta^r & U^{-1}\sin\delta^r \\ 0 & 0 & -U^{-1}\sin\delta^r & U^{-1}\cos\delta^r \end{pmatrix},$$

where $U = exp\left(-i\frac{2\pi}{\lambda}n_j d_j\right)$, $\delta^i = \delta^r = -\frac{\pi n_j Q_j d_j}{\lambda}m_z$.

The level of EAR to the magnetic material is optimised depending on the thicknesses of the top phase-compensation and bottom phase-matching SiO_2 layers, h_1 and h_2, respectively (Supplementary Fig. 5a). For the AlO_x/Co/Pt film, we calculated the non-MO reflection amplitude $|r_{xx}|$, MO reflection amplitude $|r_{xy}|$, and Kerr amplitude $|r_{xx}|/|r_{xy}|$ as a function of h_1 and h_2 (Supplementary Figs. 5b-5d). Here, the wavelength (λ) of the incident light is 660 nm. Since SiO_2 is transparent at the wavelength, the conditions of EAR repeatedly appear with a period of $\lambda/2n$ as the thickness of the phase-compensation and phase-matching layers. The refractive index (n) of SiO_2 is 1.47 and the periodicity is ~226 nm. We note that the condition of minimizing the non-MO reflection is considerably consistent with that of maximizing MO activity, resulting in two-fold enhancement of Kerr amplitude. We employed the EAR condition obtained when h_1 and h_2 are 265 and 113 nm, respectively, (indicated by the white dot). Note that, the precisely engineered EAR condition is (h_1, h_2) = (261 nm, 116 nm), which results extremely small reflectance ~6.04×10^{-6}%, and giant MOKE angle ~88.4° (Supplementary Figure 6).

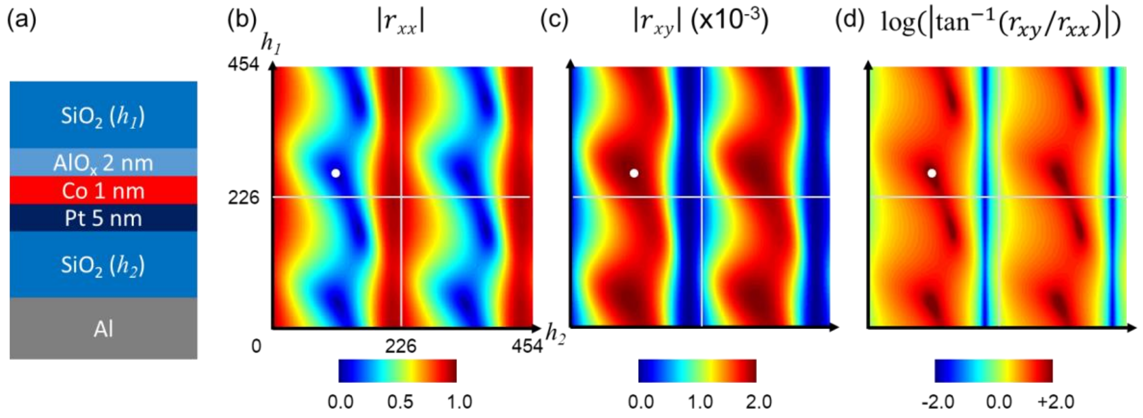

Supplementary Figure 5 (a) Schematic of the multi-layer structure for EAR. Calculated (b) non-MO reflection amplitude $|r_{xx}|$, (c) MO reflection amplitude $|r_{xy}|$, and (d) logarithmic Kerr amplitude $\log(|\tan^{-1}(r_{xy}/r_{xx})|)$ depending on the thickness of the top phase-matching and bottom phase-compensation SiO_2 layers. The white dot indicates the condition employed in this research.

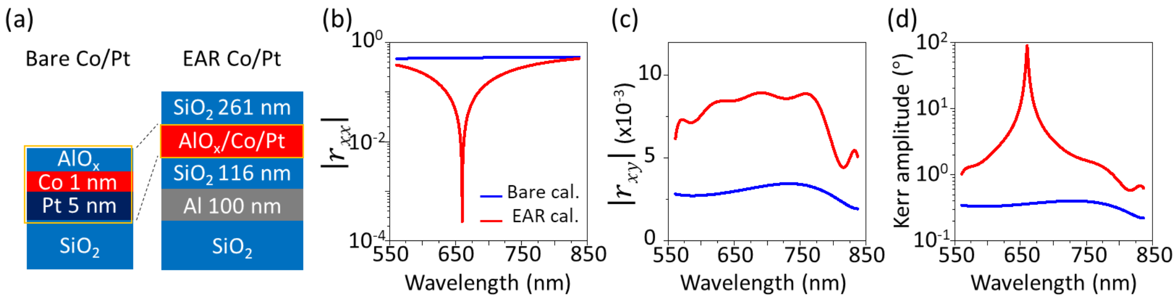

Supplementary Figure 6 (a) Schematic of the Bare Co/Pt layer and precisely engineered EAR Co/Pt layer. **(b-d)** Measured spectra of the non-MO reflection amplitude $|r_{xx}|$, MO reflection amplitude $|r_{xy}|$, and Kerr amplitude from the bare and EAR Co/Pt layers.

Supplementary Note 3. Experimental tolerance of the EAR platform to the uncertainties of layer thickness

We experimentally verified the tolerance of our EAR platform to the uncertainties of layer thickness. Employing spectroscopic ellipsometry, we measured the thicknesses of the top and bottom SiO_2 layers and the AlO_x/Co/Pt film at nine different positions on the 10 mm × 10 mm sample (Supplementary Fig. 7(a) and 7(b)). Here, we considered the AlO_x/Co/Pt film as a homogeneous medium with a complex refractive index of 2.25+3.61i and evaluated the effective thickness of the magnetic layer. Due to the non-uniformity of fabrication processes, the thicknesses of the layers change depending on the position, as shown in Supplementary Fig. 7(c). The thicknesses of the top and bottom SiO_2 layers vary from ~286.6 to ~290.0 nm and from ~111.1 to 114.1 nm, respectively, supporting nine different combinations. On the other hand, the effective thickness of the AlO_x/Co/Pt film changes only from ~9.5 to ~10.0 nm, which provided us with an appropriate condition to examine the EAR platform's tolerance to the uncertainties of the SiO_2 layers independently. We then measured the non-MO reflection coefficient and Kerr amplitude at the same positions, as shown in Supplementary Fig. 7(d) and 7(e). Despite the uncertainties of the SiO2 layers of 3~4 nm, the non-MO reflection was suppressed down to 0.03~0.045, corresponding to the reflectance of only 0.09~0.2%, around the target wavelength (660 nm), and the enhancement of the Kerr amplitude of 35~50 times larger than that of the bare Co/Pt film was achieved.

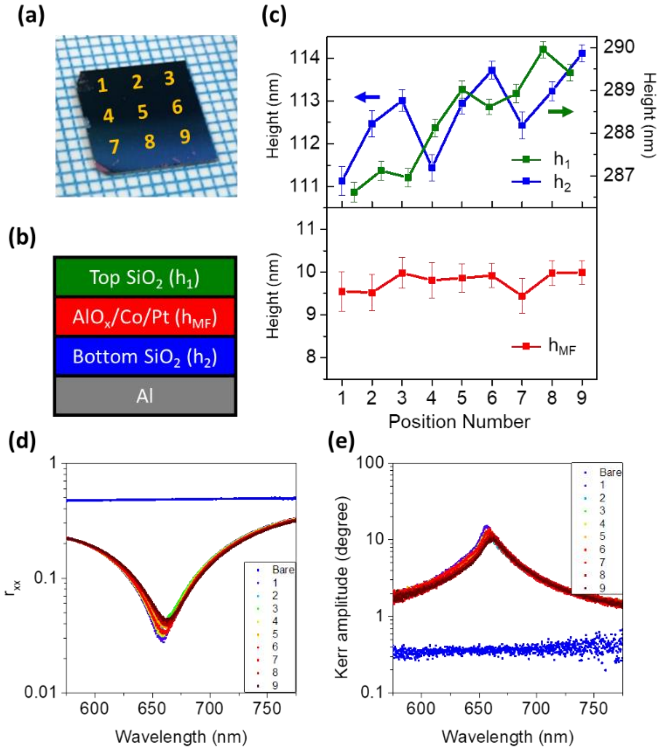

Supplementary Figure 7 Tolerance of the EAR platform to the uncertainties of layer thickness. **(a)** Camera image of the EAR Co/Pt sample. The digits indicate the nine positions where spectroscopic ellipsometry measures the layer thicknesses. **(b)** Schematic of the measured EAR Co/Pt sample. **(c)** Measured thicknesses of the top and bottom SiO_2 layers (h_1 and h_2) and measured effective thickness of the AlO_x/Co/Pt magnetic film (h_{MF}) depending on the position. **(d, e)** Measured spectra of the reflection amplitude and Kerr amplitude in the log scale.

Supplementary Note 4. Quadratic relation between the MOKE intensity and magnetisation

We consider a vertically magnetised medium of which the magnetisation is saturated to the +*z* direction. Under the x-polarised incidence with an electric amplitude E_0, the reflected electric field is given as

$$\begin{aligned} E_x &= r_{xx}E_0 \\ E_y &= r_{xy}E_0 \end{aligned} \quad \text{(Fully magnetised)}$$

Here, r_{xx} and r_{xy} are the non-MO and MO reflection coefficients of the saturated magnetic medium. If the medium is partially magnetised with a magnetisation *m* normalised to the saturation magnetisation, the non-MO reflection can be described by mr_{xy}. The value of *m* varies from −1 (fully magnetised to the **–z** direction) to +1 (fully magnetised to the **+z** direction). In MOKE microscopy, *m* corresponds to the normalised net vertical magnetisation of the magnetic domains within the resolution area of the objective. The reflected electric field from a partially magnetised medium is then described as

$$\begin{aligned} E_x &= r_{xx}E_0 \\ E_y(m) &= mr_{xy}E_0 \end{aligned} \quad \text{(Partially magnetised)}$$

The MOKE intensity (I_{MOKE}) is measured through an analyser (polariser) of angle *δ*. When we consider an actual analyser with a finite extinction efficiency *η*, the electric field components parallel and perpendicular to the optical axis of the analyser are given as

$$\begin{aligned} E_{\parallel}(m) &= |r_{xx}|E_0 \sin(\delta) + m|r_{xy}|e^{i\phi}E_0\cos(\delta) \\ E_{\perp}(m) &= \frac{1}{\sqrt{\eta}}\left[|r_{xx}|E_0\cos(\delta) - m|r_{xy}|e^{i\phi}E_0\sin(\delta)\right] \end{aligned}$$

Here, ϕ is the relative phase difference between the non-MO and MO reflection. Finally, the MOKE intensity is calculated as

$$\begin{aligned} I_{\mathrm{MOKE}}(m) &= |E_{\parallel}(m)|^2 + |E_{\perp}(m)|^2 \\ &= |E_0|^2\left[\left||r_{xx}|\sin(\delta) + m|r_{xy}|e^{i\phi}\cos(\delta)\right|^2 + \eta^{-1}\left||r_{xx}|\cos(\delta) - m|r_{xy}|e^{i\phi}\sin(\delta)\right|^2\right] \end{aligned} \quad \text{(S1)}$$

The obtained equation realised that the MOKE intensity has a quadratic dependence on the magnetization *m*. In conventional MOKE measurements, the MO reflection amplitude $|r_{xy}|$ is too small to extract the magnetisation from the measured MOKE intensity precisely. On the other hand, in the EAR MOKE microscopy, the MO reflection amplitude is comparable to the non-MO reflection amplitude. Combined with the large MO reflection, the use of a large analyser angle significantly increases the contribution of the terms, $m|r_{xy}|e^{i\phi}\sin(\delta)$ and $m|r_{xy}|e^{i\phi}\cos(\delta)$, to the MOKE intensity. Thus, we can accurately extract the magnetisation from the measured MOKE intensity with the help of the quadratic equation. If we consider the ideal case of $\eta \to \infty$, the MOKE intensity is simplified as below.

$$I_{\mathrm{MOKE}}^{\mathrm{ideal}}(m) = \lim_{\eta\to\infty} I_{\mathrm{MOKE}}(m) = |E_0|^2|r_{xx}\sin(\delta)|^2\left[1 + 2m\left|\frac{r_{xy}}{r_{xx}}\right|\cos(\phi)\cot(\delta) + m^2\left|\frac{r_{xy}}{r_{xx}}\right|^2\cot(\delta)^2\right] \quad \text{(S2)}$$

Supplementary Figure 8 shows the calculated MOKE intensity as a function of the magnetisation for the bare Co/Pt and EAR Co/Pt films. The MOKE intensity from the EAR Co/Pt film changes much larger and more sensitively than that from the bare Co/Pt film. Also, as the analyser angle increases, the relation of the MOKE intensity and magnetisation becomes more linear. In the EAR MOKE imaging (Fig. 3 in the main text), we employed an analyser angle of 10° for a high visibility.

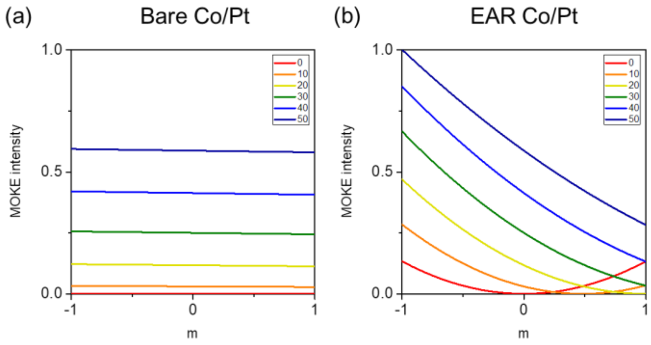

Supplementary Figure 8 Calculated MOKE intensity depending on the magnetisation in the (a) bare and (b) EAR Co/Pt films. Here, the extinction efficiency of the analyser is 10^3.

Supplementary Note 5. Visibility of MOKE microscopy

The visibility of MOKE measurement is defined as the ratio between the difference and sum of the MOKE intensities from two fully magnetised media with opposite magnetisations ($m = +1$ and $m = -1$). Employing the quadratic equation (Eq. S1), the MOKE visibility can be calculated as

$$V_{\mathrm{MOKE}} = \frac{I_{\mathrm{MOKE}}(m=+1) - I_{\mathrm{MOKE}}(-1)}{I_{\mathrm{MOKE}}(+1) + I_{\mathrm{MOKE}}(-1)} = \frac{(1-\eta^{-1})\left|\frac{r_{xy}}{r_{xx}}\right|\sin(2\delta)}{\left(1+\left|\frac{r_{xy}}{r_{xx}}\right|^2\right) - (1-\eta^{-1})\left(\cos^2(\delta) + \left|\frac{r_{xy}}{r_{xx}}\right|^2\sin^2(\delta)\right)} \quad \text{(S3)}$$

When the magneto-optic reflection (r_{xy}) is smaller than the non-magneto-optic reflection (r_{xx}), the visibility of MOKE measurement (V_{MOKE}) can be approximated as

$$V_{\mathrm{MOKE}} \cong \frac{(1-\eta^{-1})\sin(2\delta)}{1-(1-\eta^{-1})\cos^2(\delta)}\left|\frac{r_{xy}}{r_{xx}}\right| = \frac{(1-\eta^{-1})\sin(2\delta)}{1-(1-\eta^{-1})\cos^2(\delta)}|\tan(\theta_K + i\epsilon_K)|$$

The visibility of MOKE measurement depends on not only the optical angle (δ) but also the extinction efficiency (η) of the employed analyser. Using Equation S3, we calculated the visibility of MOKE microscopy in Figs. 4b and 4c in the main manuscript. Due to the extremely-small ratio of the MO reflection amplitude to the non-MO reflection amplitude, $|r_{xy}|/|r_{xx}|$, conventional MOKE microscopy can have a high visibility of >0.1 only when the extinction efficiency of the analyser is almost infinite. The actual high-quality polarisers (Wollaston prism or Glan-Thompson polarizer) with an extinction efficiency of ~10^5 support a visibility only ~0.047 in the bare Co/Pt film employed in this research (Fig. 3b in the main manuscript). On the other hand, EAR MOKE measurement can support high visibility of even up to 0.46 in maximum and >0.10 over a range of the analyser angle from 2.5° to 78° even though using a low-extinction analyser (η = 10^2), as shown in Fig. 3c in the main manuscript.

Supplementary Note 6. Magnetic domain imaging on the bare Co/Pt and Pt/Co/Pt/Ta films

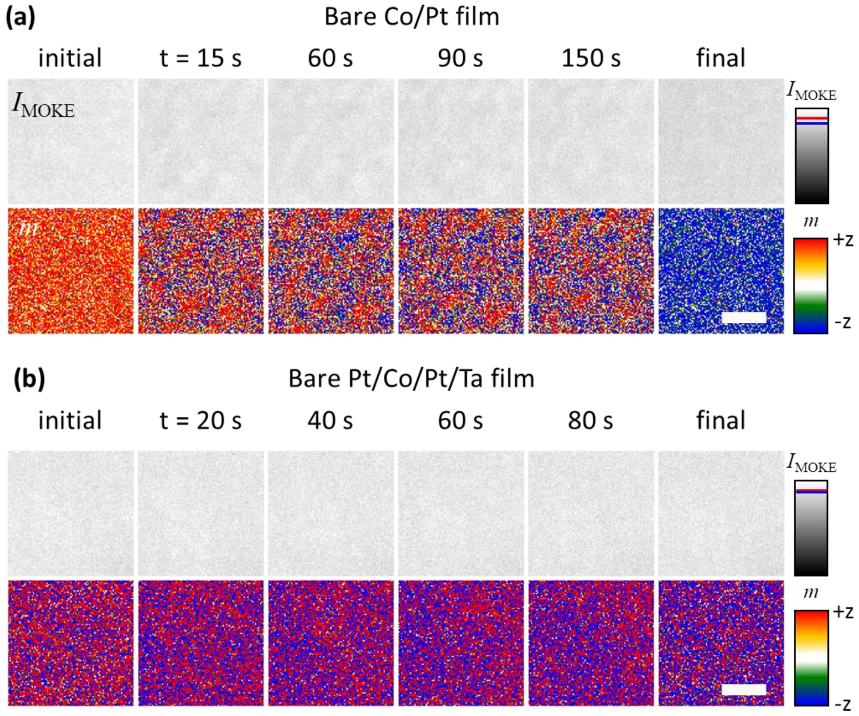

Supplementary Figure 9 Images of the measured MOKE intensity (I_{MOKE}) and extracted magnetisation (m) of magnetic domain reversal, for the bare Co/Pt **(a)** and bare Pt/Co/Pt/Ta **(b)** films. The red and blue lines in the colour bar of I_{MOKE} indicate the levels of $I_{MOKE}(+\mathbf{M})$ and $I_{MOKE}(-\mathbf{M})$. Scale bar, 3 μm. The angle and extinction efficiency of the analyser are 10° and ~10^3, respectively, which are identical to the conditions used in the results of Figs. 3d and 3e in the main manuscript. Due to the extremely-small Kerr amplitude, the visibility of MOKE intensity measurement is only 0.025 and 4.6×10^{-4} for the bare Co/Pt and Pt/Co/Pt/Ta films, respectively.

Supplementary Note 7. Avoidance of thermal effects on the magnetic domains

The 1-nm-thin Co medium exhibits the ferromagnetic ordering of the vertical magnetisation below the critical temperature (T_c). The power of the incident light should be low enough not to heat the magnetic film and influence the ferromagnetic ordering. We examined the behaviour of magnetic domain reversal depending on the power of the incident light to the EAR Co/Pt film. The incident light with a power below ~200 μW does not change the temporal behaviour of magnetic domain reversal and the final MOKE intensity (Supplementary Fig. 10a). On the other hand, the incident light of >200 μW shows notable heating effects; the speed of magnetic domain reversal becomes faster as the incident power increases (Supplementary Fig. 10b). In the measurements of Fig. 4 in the main text, we set the incident power to 110 μW that is low enough to avoid the thermal effects but high enough to employ almost the full dynamic range of the photoreceiver.

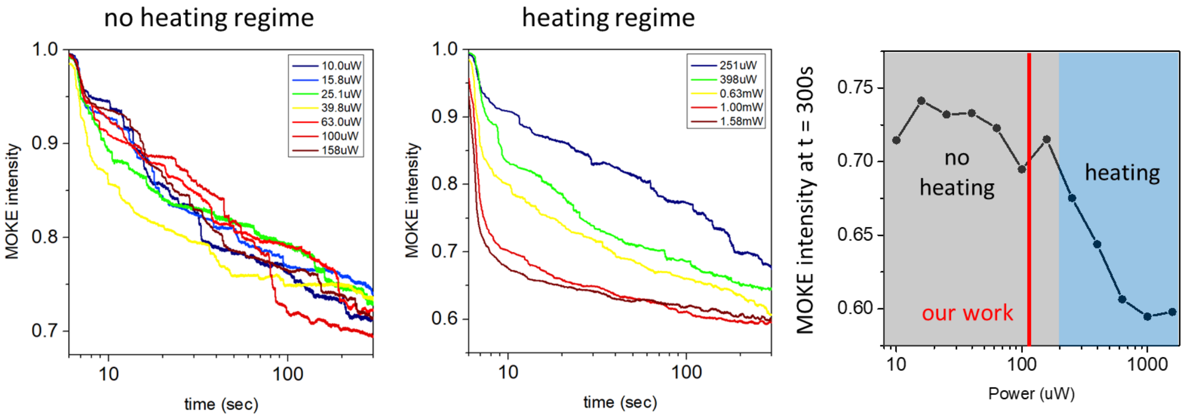

Supplementary Figure 10 Measured temporal behaviour of magnetic domain reversal of the EAR Co/Pt film in (a) unheated and (b) heated regimes. (c) Final (t = 300s) MOKE intensity depending on the incident power.

Supplementary Note 8. Detection of a single Barkhausen jump event

In our measurement setup, the output power fluctuation of the employed laser diode dominates in the signal fluctuation. The intrinsic electric noise fluctuation of the photodetector (Newport 2151 fW-detector) is smaller than the resolution of the employed analog-to-digital (AD) converter (National Instruments PCI-6111). In Fig. S11, which shows the detection of a single Barkhausen jump event in the EAR Co/Pt film, we can also identify the standard deviation of the signal fluctuation (δI_{fluc}) and the resolution of the AD converter (δI_{ADC}). The signal fluctuation, which cannot be observed when the laser is turned off, is ~1.3 times larger than the resolution of the AD converter. Here, the signal fluctuation corresponds to the area of ~35.8^2 nm^2 (=1.28×10^{-3} μm^2). In Fig. 4 in the main manuscript, we collected the stepwise signal changes by Barkhausen jumps larger than the signal fluctuation. As an example, the stepwise signal change (δI) of the single Barkhausen jump event in Fig. S11 corresponds to the area of ~69.7^2 nm^2. Meanwhile, the electric signal change corresponding to the optical diffraction limit, $(\lambda/2)^2$, is about 568 mV, which significantly larger than δI_{fluc} and δI_{ADC}.

We clarify that the accuracy of measuring the difference between the averaged values of each step of the stepwise behaviour is given by the resolution of the employed analog-to-digital (AD) converter. As shown in Supplementary Figure 11, despite the signal fluctuation, the averaged value of each step rarely changes except for the Barkhausen jump event. Thus, we can determine the difference between the two steps of the Barkhausen jump within the resolution of the AD converter. The resolution of the AD converter can be translated to the experimental uncertainty in the binning process for the statistical analysis of the Barkhausen jumps and determines the error bars in Figs. 4g and 4h of main manuscript. To avoid any possible effect of the digitization on the power-law analysis, we employed the data points apart from the minimal size of measurable areas (σ) farther than the resolution of the AD converter. We note that the signal fluctuation level was employed as the minimal size of measurable areas in a conservative manner which the signal fluctuation is mainly comes from the output power fluctuation of the employed laser diode.

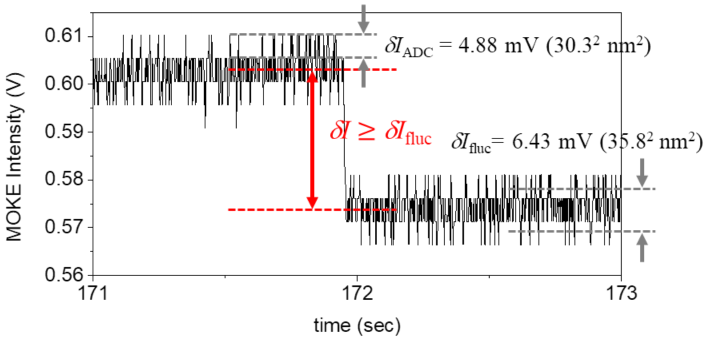

Supplementary Figure 11 Detection of a single Barkhausen jump event in the EAR Co/Pt film and identification of the signal fluctuation (δI_{fluc}) and the resolution of the AD converter (δI_{ADC}).

Supplementary Note 9. Electric voltage difference from the detector between up- and down-saturated magnetisation

Supplementary Figure12 shows typical raw data of the measured electric voltage from the detector between up- and down-saturated magnetisation for the bare Co/Pt and EAR Co/Pt films. Here, for a clear comparison, we controlled the electric voltage of up-saturated magnetisation to be the same in both cases, but the visibility does not depend on the signal intensity. The EAR Co/Pt film produces the electric voltage difference from the detector of ~7.93 V, which is ~15.86 times larger than that of the Co/Pt film (only ~0.50 V). In the bare Co/Pt film, the significant non-MO background reflection causes the high voltage signal at the down-saturated magnetisation.

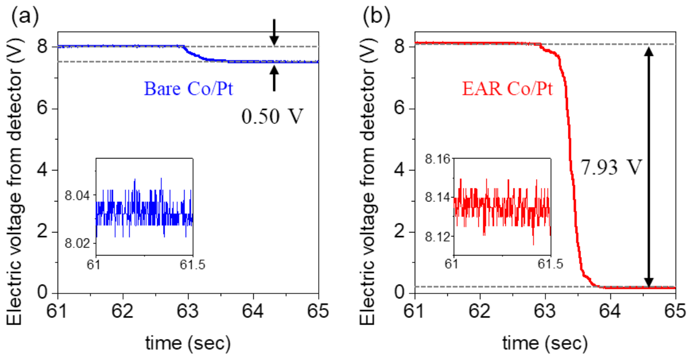

Supplementary Figure 12 Typical raw data of the measured electric voltage from the detector between up- and down-saturated magnetisation of the bare Co/Pt **(a)** and EAR Co/Pt **(b)** films. (inset) Signal fluctuation in the measurement.

Supplementary Note 10. Characterisation of the photodetector for Barkhausen jump measurement

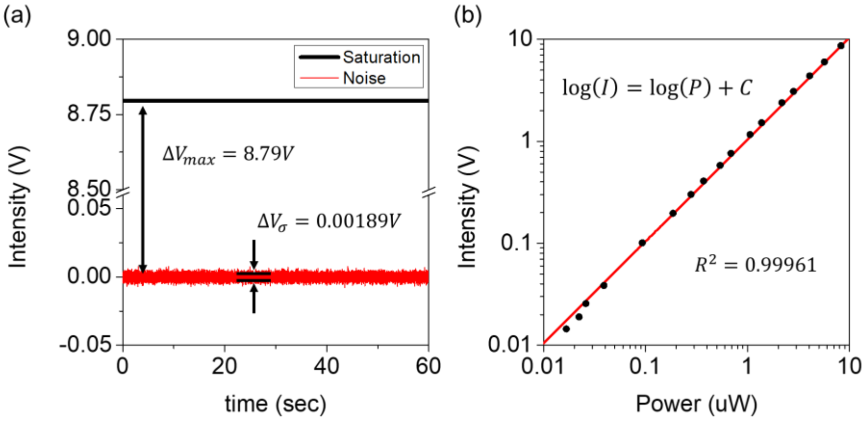

Supplementary Figure 13 (a) Real-time measurement of the saturation level (black) and noise fluctuation (red) of the employed photodetector (Newport 2151 fW-detector and National Instruments PCI-6111 analog-to-digital converter). (b) Optical power dependent response of the photodetector (log-log scale).

Reference

[1] Huang, B. *et al.* Layer-dependent ferromagnetism in a van der Waals crystal down to the monolayer limit. *Nature* **546**, 270–273 (2017).

[2] Zhang, Z. *et al.* Direct Photoluminescence Probing of Ferromagnetism in Monolayer Two-Dimensional CrBr 3. *Nano Lett.* **19**, 3138–3142 (2019).

[3] Lee, J. U. *et al.* Ising-Type Magnetic Ordering in Atomically Thin FePS3. *Nano Lett.* **16**, 7433–7438 (2016).

[4] Chumak, A. V., Vasyuchka, V. I., Serga, A. A. & Hillebrands, B. Magnon spintronics. *Nat. Phys.* **11**, 453–461 (2015).

[5] Åkerman, J. Toward a universal memory. *Science* **308**, 508–510 (2005).

[6] Kent A. D., Worledge D. C., A new spin on magnetic memories. *Nat. Nanotech.* **10**, 187-191 (2015)

[7] T. Izuhara, et. al., Direct wafer bonding and transfer of 10-μm-thick magnetic garnet films onto semiconductor surfaces, *Appl. Phys. Lett.* **76** 1261-1263 (2000)

[8] S. Ghosh, et. al., Ce:YIG/Silicon-on-Insulator waveguide optical isolator realized by adhesive bonding, *Opt. Express* **20** 1839-1848 (2012)

[9] You, C. Y. & Shin, S. C. Generalized analytic formulae for magneto-optical Kerr effects. *J. Appl. Phys.* **84**, 541–546 (1998).
